# New Experimental Approach for the Proper Consideration of Stagnant and Diffuse Layer Conductivity in the Zeta Potential Determination

**DOI:** 10.1021/acs.langmuir.4c04456

**Published:** 2025-02-19

**Authors:** Matthias Frangenberg, Annette M. Schmidt, Jan Wilkens

**Affiliations:** a Faculty of Applied Natural Sciences, TH Köln − University of Applied Sciences, Leverkusen D-51379, Germany; b Department of Chemistry, Institute of Physical Chemistry, University of Cologne, Cologne D-50939, Germany

## Abstract

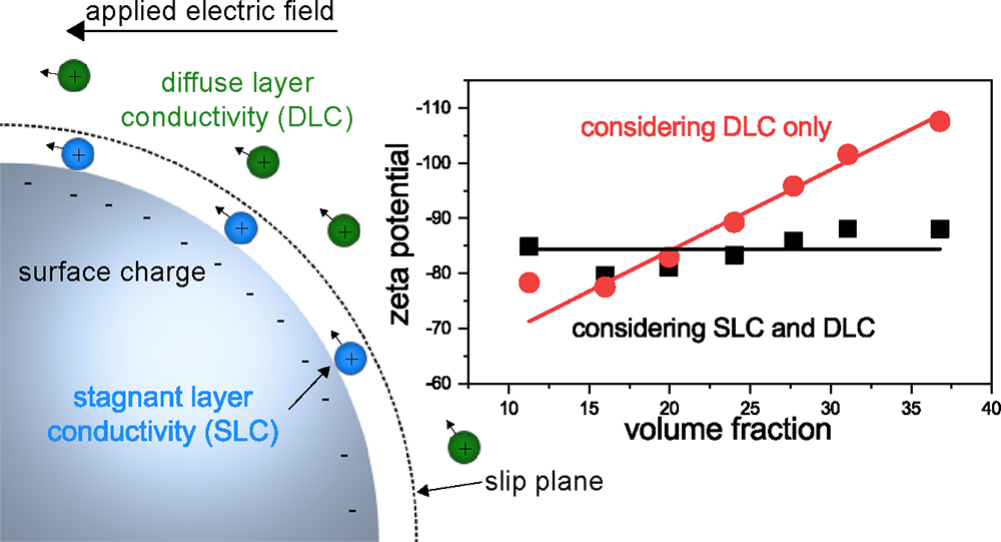

Accurate determination of the zeta potential in colloidal dispersions often requires consideration of the relaxation effect, which is associated with the polarization of the electrical double layer and the surface conductivity. In this study, we pursue a new approach that combines conductivity measurements of the dispersion and dispersion medium with the electroacoustic and electrophoretic zeta potential determination. The conductivity data are analyzed with the Maxwell–Wagner–O’Konski theory, providing the Dukhin number. Zeta potentials of highly concentrated polymer dispersions were determined using the colloid vibration current (CVI) method and compared with those obtained by electrophoretic light scattering (ELS) in diluted dispersions. In both cases, the relaxation effect was now taken into account on the basis of the experimentally determined Dukhin number. The evaluation of the Dukhin numbers revealed significant surface conductivity for all investigated polymer dispersions. In addition, it was often found that not only the diffuse layer but also the stagnant layer contributes considerably to the surface conductivity. Proper consideration of both effects is essential for the reliable determination of the zeta potential, as otherwise inconsistencies can be observed in the evaluated data. Moreover, we have validated for the first time that the advanced CVI theory takes the effect of surface conductivity properly into account for a wide range of particle volume fractions. These values agree well with those obtained by the ELS method using the Dukhin–Semenikhin theory or a modified theory of Ohshima, Healy, and White. This study thus shows that the Dukhin number can serve as a key parameter to reliably connect conductivity and electrophoretic and electroacoustic experiments.

## Introduction

The zeta potential is an important parameter that provides valuable insight into the electrical properties of charged interfaces, i.e., the constitution of the electrical double layer (EDL). It depends on the nature of the particles and the dispersion medium, e.g., the pH value, ionic strength, and chemical composition. A well-established method for its determination is electrophoretic light scattering (ELS). Usually, measurements are performed with diluted dispersions to avoid errors due to multiple light scattering.

Advances in measurement technology and theoretical foundations have made it possible to use the electroacoustic method as an alternative for the determination of the zeta potential in concentrated systems. Electroacoustic methods exist in two implementations depending on the driving force moving the particles: electrokinetic sonic amplitude (ESA) and colloid vibration current (CVI) setups.^1,2^ There are commercial devices available that are based on either the ESA or CVI method. Both electrophoretic and electroacoustic methods nowadays reach a high level of maturity, which is reflected in the existence of International Standards.^3−5^

An experimentally measured output of ELS studies is the electrophoretic mobility. The corresponding equivalent in electroacoustics is the so-called dynamic electrophoretic mobility. These quantities are closely related to the zeta potential and can be converted into each other using various theories. Standard theories such as the Debye–Hückel,^6^ the Henry,^7^ or the Helmholtz–Smoluchowski theory^8^ are easy to use, but their application is subject to several restrictions. For example, they are only valid if the ratio of the particle radius to the reciprocal Debye–Hückel parameter, which is a characteristic length of the EDL, is within a certain range. Moreover, the zeta potential must be quite low, as these theories disregard the relaxation effect, i.e., the impact of surface conductivity and polarization of the EDL on the electrophoretic mobility. Advanced theories attempt to address these limitations. However, they require knowledge of more input parameters than the classical ones. This may explain why the relaxation effect is often neglected in zeta potential determination.

A comprehensive overview concerning the influence and consideration of the surface conductivity and the polarization of the EDL can be found in various review articles, for example, refs (9−11), and in an IUPAC Technical Report.^12^ Surface conductivity arises from ions that are located in both parts of the EDL, namely in the diffuse layer and in the hydrodynamically immobile stagnant layer behind the slip plane. Several studies deal with the contribution of both conductivities on zeta potential determination.^13−35^ Although the potential impact of stagnant layer conductivity (SLC) is now well known, many publications focus solely on the contribution of the diffuse layer or neglect the surface conductivity completely. This may lead to an underestimation of the zeta potential. Therefore, it is recommended to check the boundary conditions and carefully choose an appropriate theory for the conversion of the primary measurement signal into zeta potential.^12^

Surface conductivity cannot be measured directly, but various investigation methods can be used to estimate this quantity or at least give an indication of its presence. The authors of the IUPAC Technical Report recommend, for example, to measure the electrophoretic mobility as a function of the electrolyte concentration. Significant surface conductivity can then be recognized by an extremum at low electrolyte concentrations. They also refer to the possibility of determining the Stern potential by means of particle charge titration and comparing it with the measured zeta potential. Deviations between these values then indicate that the surface conductivity was not correctly taken into account in the calculation of the zeta potential.^12^ Moreover, the impact of surface conductivity is also reflected in the electrical conductivity of a dispersion. Several theories have been developed in the past to describe this relationship, e.g. by Maxwell, Wagner, and O’Konski (MWO),^36−38^ Ohshima,^39^ or Carrique et al.^40,41^ International Standards therefore recommend to measure the electrical conductivity of the dispersion and the dispersion medium at different volume fractions of dispersed particles. From these data, the Dukhin number, which is a measure of the surface conductivity contribution to electrokinetic phenomena, can then be extracted using the MWO theory.^4,42^ This procedure offers some advantages. First, the Dukhin number determined in this way covers the contributions of both stagnant and diffuse layers. Second, this parameter can be directly used in the evaluation of electrophoretic and electroacoustic measurement data by applying certain advanced theories, but to the best of our knowledge, this approach has not yet been carried out.

Among the advanced theories, the analytical expression of Ohshima, Healy, and White (OHW theory)^43^ is broadly recognized, as it leads to exact solutions although it does not use numerical methods. However, it should be noted that this theory only takes into account the diffuse layer conductivity (DLC) and cannot process an experimentally determined Dukhin number. The full Dukhin–Semenikhin theory (DS theory),^44,45^ on the other hand, does not have these limitations but requires knowledge of the Stern potential and ion diffusion in the stagnant layer, which makes its practical application a bit more difficult. Dukhin and Semenikhin also developed a simplified version of their theory^45,46^ that eliminates these special requirements. However, both versions of DS theory have rarely been applied so far,^47−56^ and there is a lack of studies demonstrating their performance when using the experimentally determined Dukhin number.

Over the last decades, electroacoustics has proven to be a reliable tool for determining the zeta potential of inorganic dispersions, even at high particle volume fractions.^57^ However, only a few studies have so far examined the electroacoustic behavior of polymer dispersion.^15,58−68^ In-depth investigations of polymer latices would therefore be useful to better understand the capabilities and limitations of this measurement method. Due to this knowledge gap, one aim of the present study is a detailed investigation of the applicability and requirements of electroacoustic measuring methods for determining zeta potential of concentrated polymer dispersions.

We have already reported our findings using the Helmholtz–Smoluchowski and Henry frameworks in a recent publication.^68^ In this study, we focus on the correct consideration of the relaxation effect and the surface conductivity. We present a new experimental approach that combines the Dukhin number determined by MWO theory with the electroacoustic and electrophoretic determination of the zeta potential.

The zeta potentials of concentrated polymer dispersions are determined using the CVI method in combination with the advanced CVI theory, which is capable of using the Dukhin number to account for the relaxation effect. Consequently, contributions of both diffuse layer and stagnant layer are reflected in the calculated zeta potential. In order to perform an “electrokinetic consistency test”, measurements of the zeta potential are also carried out on diluted samples using the ELS method. On the one hand, we evaluate these data with an analytical expression derived by Ohshima, Healy, and White that considers the relaxation effect only on the basis of the surface conductivity in the diffuse layer. We have additionally modified this expression to consider the influence of surface conductivity by using the Dukhin number. On the other hand, we also apply the Dukhin–Semenikhin theory in the full and simplified version, which takes both SLC and DLC into account if the Dukhin number is properly determined. This allows a comprehensive comparison of the two experimental approaches and the evaluation theories used. In addition, we obtain information on the significance of the surface conductivity contribution in the stagnant layer.

## Theory

### Electrical Double Layer and Zeta Potential

The electrokinetic behavior of charged colloidal particles dispersed in an electrolyte solution is strongly affected by the distribution of these ions in the vicinity of the particle. This arrangement is called electrical double layer (EDL), and various models can be used to describe its structure in varying degrees of complexity. The Gouy–Chapman–Stern model is rather simple and is often adopted for describing many electrokinetic phenomena. It postulates formation of two layers at the electrically charged particle interface. The inner layer, called the Stern layer, consists of a compact layer of adsorbed ions that are strongly bound to the interface. The outer, diffuse layer encompasses the ions that are distributed diffusely within the dispersion medium due to thermal motion. Compared to the bulk medium, the diffuse layer and the Stern layer have an excess electric charge with a sign that is opposite to the sign of the surface charge. This allows the entire EDL structure to be neutral.

An external electric field or mechanical force can provoke a tangential flow of the dispersion medium relative to the charged surface. The velocity decreases to zero at a certain distance from the surface, which is referred to as the hydrodynamic slip or shear plane. The layer that extends from the surface to the slip plane is known as the stagnant layer. Although it is hydrodynamically immobile, the electrolyte ions in this layer can still conduct electricity. It is worth mentioning that the stagnant layer and the Stern layer do not necessarily coincide.^12^

The electric potential of the charged interface in EDL is called surface potential ψ_0_, and that of the external boundary of the Stern layer is referred to as Stern potential ψ_d_. The electric potential at the slip plane in the EDL is defined as the zeta potential ζ. Its value depends on the chemical composition of the charged interface, as well as the electrolyte content and chemical composition of the dispersion medium. Typically, it decreases with increasing electrolyte concentration due to the compression of the diffuse layer.

### Surface Conductivity

The total ion concentration in the EDL is higher than in the bulk medium, resulting in an excess electric conductivity known as surface conductivity *K*^σ^. It contributes together with the conductivity of the dispersion medium *K*_m_ to the total conductivity of the dispersion *K*_s_.

The significance of surface conductivity *K*^σ^ compared to the medium conductivity *K*_m_ can be expressed by the dimensionless Dukhin number *Du*, where *a* is the particle radius:
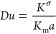
1

It is also important to note that the surface conductivity contains contributions of ions in the diffuse layer (*K*^σd^) as well as mobile ions in the stagnant layer (*K*^σs^). Therefore, the surface conductivity and the Dukhin number must be expressed by the sum of both contributions:^12^
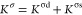
2
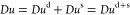
3

The DLC *K*^σd^ is also known as Bikerman surface conductivity and can be calculated for a symmetrical *z*–*z* electrolyte in the case of κ*a* ≫ 1 using eq 4.^12^ It should be noted that this calculation requires knowledge of the correct zeta potential.
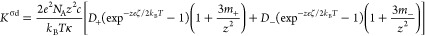
4

Here, *D*_±_ is the diffusion coefficient of cations (+) and anions (−). It can be calculated according to the Nernst–Einstein relation.
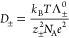
5*m*_±_ is the dimensionless ion mobility of the electrolyte ions
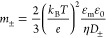
6and κ is the Debye–Hückel parameter
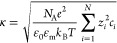
7where *e* is the elementary charge, *N*_A_ is the Avogadro constant, *z*_*i*_ is the valence and *c*_*i*_ is the concentration of the electrolyte ions, *k*_B_ is the Boltzmann constant, *T* is the thermodynamic temperature, and Λ_±_^0^ is the limiting molar conductivity of electrolyte ions at infinite dilution.

Often, κ cannot be calculated according to eq 7, since the exact composition of the dispersion medium is unknown. Therefore, its value can be approximated utilizing the conductivity of the dispersion medium *K*_m_ and eq 8.^4^ For the effective diffusion coefficient *D*_eff_, the average value of the presumed electrolyte should be used. This parameter also accounts for the greatest uncertainty in this equation due to an unknown electrolyte composition. However, *D*_eff_ usually falls within a predictable range, as the diffusion coefficients of most ions in aqueous solutions at room temperature typically span from 0.6 × 10^–9^ to 2 × 10^–9^ m^2^·s^–1^. The square root of *D*_eff_ further reduces the uncertainty in the estimation of the Debye–Hückel parameter according to eq 8.^2^
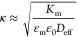
8

Finally, to ease the calculation of *K*^σd^ or *Du*^d^, a simplification is often made. Assuming *m*_+_ = *m*_–_ = *m*, one obtains^12^
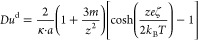
9

This equation can be approximated well for large zeta potentials (say ζ > 50 mV) as follows:
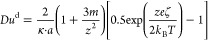
10

As already mentioned, the Bikerman theory only accounts for the DLC, i.e., *K*^σd^. The second contribution to the surface conductivity is provided by the SLC, i.e., *K*^σs^. A direct measurement of this parameter is not yet possible. Therefore, it is usually determined by means of eq 2 when *K*^σ^ and *K*^σd^ are known. Accordingly, *K*^σ^ or *Du* has to be measured.

### Experimental Determination of the Dukhin Number

For a detailed characterization of the surface conductivity, it is useful to know the Dukhin number *Du*. According to the recommendation in International Standard ISO 13099, Part 1, Appendix B.3,^4^*Du* can easily be determined using conductivity measurements. This procedure includes measurement of the conductivities of the dispersion (*K*_s_) and the dispersion medium (*K*_m_). Such measurements should be performed at various particle volume fractions φ. The relative conductivity *K*_s_ · *K*_m_^–1^ of a dispersion of nonconducting spherical particles can be described by the MWO theory,^36−38^ and, finally, *Du* can be calculated by least-squares fit using eq 11.
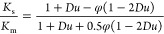
11

The Dukhin number determined experimentally in this way reflects the surface conductivity in both diffuse and stagnant layer. For the sake of distinction, we designate it with *Du*^d+s^. The contribution of the diffuse layer alone (*Du*^d^) can be calculated using eq 9. Finally, the contribution of the stagnant layer (*Du*^s^) can be determined by subtracting *Du*^d^ from *Du*^d+s^ according to eq 3.

### Advanced Zeta Potential Theory for the CVI Method

O’Brien introduced the concept of the dynamic electrophoretic mobility μ_d_ in the context of electroacoustic measurements, whose basic expressions are given in eqs 12 and 13.^69^ It relates measured electroacoustic parameters to relevant colloidal parameters, including the zeta potential ζ. Based on this pioneering concept, *A*. Dukhin and colleagues developed an advanced zeta potential theory specifically for CVI measurements.^2,70^ Their approach extends O’Brien’s framework by considering alternative cell models to account for the effects of particle–particle interactions in concentrated dispersions on the dynamic electrophoretic mobility μ_d_.^71−73^
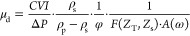
12
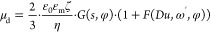
13

In eq 12, CVI is the measured colloidal vibration current amplitude, Δ*P* is the pressure difference, *A*(ω) is an instrument constant that is determined by calibration, and *F*(*Z*_T_, *Z*_s_) is a function of the acoustic impedance of the instrument transducer *Z*_T_ and dispersion *Z*_s_. The equation also accounts for the densities of the dispersion ρ_s_ and the particles ρ_p_, as well as the volume fraction of the particles φ.

Equation 13 introduces the factor *G*(*s*, φ) to consider inertial effects and the factor *F*(*Du*, ω^′^, φ) to describe electrodynamic effects (*s* and ω^′^ are factors depending on the angular frequency ω). In the advanced CVI theory, O’Brien’s original approach was adapted by refining these functions. The factor *F*(*Du*, ω^′^, φ) also accounts for the Dukhin number and accordingly for the effects of surface conductivity and EDL polarization.^74^ Therefore, accurate determination of the Dukhin number, which considers both the conductivity contributions beyond and behind the slip plane, is of particular importance in order to obtain reliable values for the zeta potential ζ.

The advanced CVI theory is applicable in cases of a thin EDL (i.e., κ*a* ≫ 1). In-depth information on this sophisticated theory and the extensive calculation of the factors *G*(*s*, φ) and *F*(*Du*, ω^′^, φ) can be found in ref (2), chapter 5.4.

### Advanced Zeta Potential Theory for the ELS Method

The renowned theory developed by O’Brien and White^75^ for electrophoresis includes the effect of surface conductivity and EDL polarization. It uses numerical calculations for the zeta potential of a spherical colloidal particle and is applicable for any range of zeta potential, arbitrary κ*a* values, and different electrolyte valences. However, it should be noted that this theory only accounts for the DLC *K*^σd^, but not for the surface conductivity of the stagnant layer *K*^σs^. Mangelsdorf and White^76^ later extended the theory of O’Brien and White to consider the effect of SLC as well. They used a dynamic Stern layer model, which refers to the adsorption of ions and their mobilities within the stagnant layer. This theory is valid just like the theory of O’Brien and White for arbitrary κ*a* values. It requires information about several parameters that describe the characteristics of the dynamic Stern layer. These input parameters can be determined, for example, by a combined analysis of electrophoretic and dielectric dispersion data.^29^

#### Theory of Ohshima, Healy, and White

As an alternative to numerical methods, several analytical expressions have been derived that approximate the relationship between the electrophoretic mobility and the zeta potential of a spherical hard particle. Usually, boundary conditions concerning the product of the Debye–Hückel parameter κ and the particle radius *a* have to be considered. For example, Ohshima, Healy, and White (OHW) derived eq 14 for a symmetrical *z*–*z* electrolyte. It is valid for κ*a* ≥ 10 with a relative error of less than 1%, regardless of the value of the zeta potential.^43^
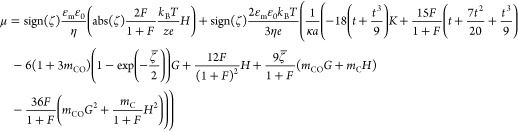
14

Here, *m*_C_ and *m*_CO_ are the dimensionless ion mobilities of the counterion and co-ion, respectively. Equations 15 to 20 provide the values for the dimensionless zeta potential ζ̅ as well as the individual functions *F*, *G*, *H*, *K*, and *t*.
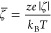
15
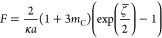
16
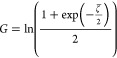
17
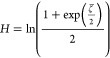
18
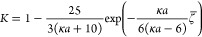
19
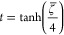
20

When using this analytical expression to calculate the zeta potential, it is important to note that it only accounts for the surface conductivity of the diffuse layer *K*^σd^ (i.e., DLC). Errors in zeta potential determination are to be expected if SLC is significant. We have therefore looked for a way to introduce the Dukhin number into the OHW theory. The combination of eq 10 with eq 16 gives eq 21, which initially takes *Du*^d^ into account. However, it also offers the possibility to consider the SLC contribution to the surface conductivity in the OHW theory on a trial basis if *Du*^d+s^ values are used for the Dukhin number instead of *Du*^d^.
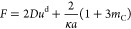
21

#### Theory of Dukhin and Semenikhin

For comparison with the OHW and advanced CVI theory, we can use the approximate eq 22 of S. Dukhin and Semenikhin (DS theory), which is valid for symmetrical *z*–*z* electrolytes.^4,44,45^ Moncho et al. showed that the relative error is low if κ*a* is larger than 25–50.^55^ It should be noted that this κ*a* limit is somewhat higher than that of the OHW theory.
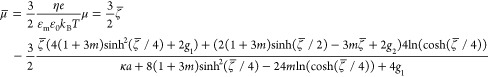
22with
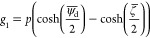
23
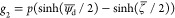
24
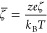
25
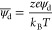
26

The ion diffusion coefficient in the boundary layer *D*_±_^s^ might be lower than its value in the bulk medium *D*_±_. This influence is therefore taken into account by the parameter *p*:
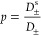
27

Equation 22 considers both the dimensionless Stern potential  and the dimensionless zeta potential ζ̅. The relationship between these variables can be established using the parameter *p* and the Dukhin number *Du*^d+s^, which covers both DLC and SLC. As can easily be seen, the first product term in the bracket of eq 28 corresponds to *Du*^d^ (cf. eq 9) and the second to *Du*^s^.
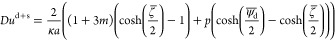
28

The DS theory also provides a simplification of eq 22, which is shown in eq 29. It is valid for  > 1.5.^45,46^ In this case, knowledge of the parameter *p* is not a prerequisite for calculating the zeta potential. For correct consideration of the surface conductivity, the Dukhin number *Du*^d+s^ should again be used in eq 29. Please note that, unlike in the original equation (e.g., eq 3.8.13 in ref (45)), we have divided by the absolute value of the dimensionless zeta potential |ζ̅|. As we will show in Discussion of Sample PVC@0.20 for the ELS Method, this is necessary to obtain the correct solution in the case of negative electrophoretic mobilities.
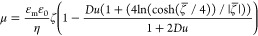
29

## Experimental Section

### Materials and Characteristic Dispersion Parameters

We examined a variety of polymer latices in this study. Each sample identifier comprises the material and particle diameter *d*_50_ in microns, i.e., material@*d*_50_. Three polyvinyl chloride (PVC) dispersions from Westlake Vinnolit GmbH & Co. KG were used: PVC@0.20, produced by emulsion polymerization; PVC@0.78 and PVC@2.10, both produced by microsuspension polymerization. A styrene–butadiene rubber, Taktene S 62 F (SBR@0.23), was supplied by ARLANXEO Switzerland S.A. Furthermore, a polyurethane dispersion (PU@0.07; synthesized as part of an unpublished master’s thesis of Christina Gassner at Heinrich Heine University Düsseldorf in 2021) and a poly(methyl methacrylate-*co*-butyl acrylate) dispersion (PBAMM@0.1; synthesized as part of an unpublished master’s thesis of Sven Kroß at TH Köln in 2021) were included in this study.

The particle densities were measured using a pycnometer, and pH values were determined using a calibrated 9615S-10D electrode from Horiba. Particle size distributions (PSDs) were determined by dynamic light scattering or laser diffraction spectroscopy, depending on the particle size. For detailed information regarding these measurements, refer to our recent publication.^68^ The resulting dispersion characteristics are summarized in Table 1 and Table S1 in the Supporting Information.

**Table 1:** Summary of Relevant Sample Characteristics^a^

sample	diluent/target concentration	ρ**_p_** in g·cm^–3^	*d*_50_ in μm	*K*_m_ in mS·cm^–1^	pH value
PVC@0.20	supernatant	1.41	0.202	1.78 ± 0.03	9.0 ± 0.1
PVC@0.78	supernatant	1.39	0.783	4.24 ± 0.37	6.8 ± 0.1
PVC@2.09	supernatant	1.41	2.086	0.69 ± 0.02	9.6 ± 0.1
SBR@0.23	10 mM KCl	0.94	0.233	1.27 ± 0.13	8.9 ± 0.1
PU@0.07	10 mM KCl	1.10	0.069	2.17 ± 0.76	8.8 ± 0.4
PBAMM@0.10	10 mM KCl	1.16	0.099	6.09 ± 2.93	8.6 ± 0.1

a The particle densities ρ_**p**_ were analyzed at 25 °C, and the particle size data *d*_50_ are based on a volume weighted particle size distribution. All particles were removed with a centrifuge prior to the measurement of the electrical conductivity *K*_m_ of the dispersion medium. For further details, refer to ref (68). Calculated standard deviations are absolute values from triplicate measurements.

### Sample Preparation

The samples have to be diluted in steps for most measurements. We used equilibrium supernatant for conducting “equilibrium dilution”.^4^ This supernatant was extracted by centrifugation. Therefore, the chemical equilibrium between the particle surface and the dispersion medium is maintained. However, supernatant was not available in some cases. We then used a solution that approximately resembles the dispersion medium. These would be “nonequilibrium dilutions”.

Different dilution protocols were used depending on the available sample quantity and the applied measurement technique. The detailed procedure can be found in ref (68), but the most important aspects will be briefly outlined here for better understanding.

#### CVI Method

All PVC samples were diluted with their equilibrium supernatant achieving true equilibrium dilution.

The SBR sample was first dialyzed and then diluted with the appropriate amount of electrolyte solution to achieve a nominal concentration of 10 mM KCl in the dispersion medium. All other samples were directly diluted to have a nominal electrolyte concentration of 10 mM KCl in the dispersion medium (cf. Table 1), neglecting the unknown initial electrolyte background. These samples may therefore deviate from equilibrium dilution.

#### ELS Method

In the case of PVC samples, the equilibrium supernatant was used for dilution, same as for CVI measurements.

All other samples were diluted using a 10 mM KCl solution. The degree of dilution of the stock dispersion is so pronounced that the influence of the original electrolyte background can be neglected.

### Determination of the Dukhin Number

In order to determine the Dukhin number *Du*, the relative conductivities *K*_s_ · *K*_m_^–1^ were determined at different particle volume fractions. The corresponding conductivities of the dispersion (*K*_s_) and the dispersion medium (*K*_m_) were measured using the conductivity electrode of the CVI measurement device DT-1202 from Dispersion Technology at 3 MHz and room temperature. Measurements were performed in triplicate, and the results were averaged. Then, we applied the Maxwell–Wagner–O’Konski (MWO) theory, as described in Experimental Determination of the Dukhin Number. The value of the Dukhin number *Du* can be computed by least-squares fit using eq 11 and fixing the correct limiting behavior *K*_s_ · *K*_m_^–1^ → 1 at vanishing volume fractions. For the sake of distinction, we designate the corresponding values with *Du*^d+s^, reflecting that surface conductivity is considered in both the stagnant and diffuse layer. The electrolyte concentrations of all samples were above the isoconductive point, as the relative conductivities *K*_s_ · *K*_m_^–1^ were less than one and the resulting *Du*^d+s^ values were below 0.5. More details about the experimental procedure and the results can be found in ref (68).

### Determination of Zeta Potential by the CVI Method

CVI measurements were performed using a DT-1202 device from Dispersion Technology Inc. at room temperature. Frequency of ultrasound was 3 MHz. We ignored contribution of ions (so-called ion vibration current) because it was negligible compared to the contribution of charged particles. To prevent settling and inhomogeneity, the samples were circulated with a peristaltic pump during measurement. Each sample was measured three times, with the results being averaged.

The determination of the zeta potential with the advanced CVI theory requires the Debye–Hückel parameter κ and the Dukhin number. Since the composition of the dispersion medium was not known, we used a method described in ISO Standard 13099, Part 1.^4^ Consequently, the value of the parameter κ was calculated with eq 8 using the conductivity data *K*_m_ of the dispersion medium given in Table 1. For the effective diffusion coefficient *D*_eff_, a value of 1.994 × 10^–9^ m^2^·s^–1^ was used, which corresponds to potassium chloride.

The software of DT-1202 has an option for calculating the Dukhin number automatically when extracting the zeta potential value from measured CVI. It employs essentially the Bikerman approach. Therefore, it only takes into account DLC, when working in automatic mode. However, the latest software version (12 m56060) now also offers the option to enter the *Du* value manually. This allows the use of a Dukhin number that correctly reflects both contributions to the surface conductivity, from diffuse and from stagnant layer.

To investigate the dependence of the zeta potential on the electrolyte concentration, KCl solutions of varying concentrations were added to the stock dispersion of the PVC@0.20 sample. A particle volume fraction of approximately 11.3 vol % was adjusted, and the actual values were determined by analyzing the dry residue. The electrolyte content of the stock dispersion was not known but was diluted during sample preparation. The respective *Du*^d+s^ values were determined from the relative conductivities *K*_s_ · *K*_m_^–1^ using eq 11. For details on the calculation of the zeta potential according to Henry’s theory, we refer to our previous publication.^68^

### Determination of Zeta Potential by the ELS Method

ELS measurements were performed using a ZetaSizer Nano ZS device from Malvern Panalytical. The equilibration time was set to 180 s and the number of runs to 50. The attenuation, measuring position, and voltage were selected automatically by the software (version: 7.13). All measurements were performed at 25 °C using a wavelength of 633 nm and folded capillary cells DTS 1070. The samples were diluted according to the protocol described in Sample Preparation. The pH values were adjusted to the same values as for the CVI measurements using 0.1 mol L^–1^ NaOH or HCl solution. To reveal multiple light scattering effects, measurements were performed at least at five different particle volume fractions. The volume fractions studied were approximately between 0.1 and 0.001 vol %.

The measured electrophoretic mobilities were converted into zeta potentials using eq 14. For most samples, the required κ values were calculated according to eq 7, assuming a concentration of 10 mM KCl (cf. Table 1) as justified by the dilution procedure. In the case of the PVC samples, the approximated κ values of the CVI measurements have to be used because information about the electrolyte composition were missing. For comparison, the ELS measurement results were also evaluated with eqs 22 and 29 using the experimentally determined Dukhin numbers *Du*^d+s^. In all cases, only values with almost identical zeta potentials were averaged (inappropriate particle concentrations were easily recognized by significantly deviating values, which would also increase the determined standard deviations).

## Results and Discussion

A systematic of symbols is used for the detailed presentation of our results, as summarized in Table 2 with a brief description. Detailed explanations are given in the subsequent sections.

**Table 2:** Reference Table of Symbols

symbol^a^	description
*Du*^d+s^	Dukhin number calculated according to eq 11 using conductivity data and MWO theory, reflecting surface conductivity in both diffuse and stagnant layer
*Du*^d^	Dukhin number calculated according to eq 9 using ζ_CVI_^d+s^ and κ*a*_CVI_, reflecting surface conductivity in the diffuse layer only
*Du*_DT_^d^	Dukhin number calculated automatically by DT-1202 software using conductivity of the dispersion *K*_s_, reflecting surface conductivity in the diffuse layer only
*Du*^s^	Dukhin number calculated by subtracting *Du*^d^ from *Du*^d+s^, reflecting surface conductivity in the stagnant layer only
ζ_CVI_^d^	zeta potential calculated according to eqs 12 and 13 from CVI data for *Du*_DT_^d^, reflecting surface conductivity of diffuse layer only
ζ_CVI_^d+s^	zeta potential calculated according to eqs 12 and 13 from CVI data for *Du*^d+s^, reflecting surface conductivity in both diffuse and stagnant layer
ζ_ELS_^d^	zeta potential calculated according to eq 14 under consideration of eq 16 from ELS data, reflecting surface conductivity of diffuse layer only (original OHW theory)
ζ_ELS1_^d+s^	zeta potential calculated according to eq 14 under consideration of eq 21 from ELS data for *Du*^d+s^, reflecting surface conductivity in both diffuse and stagnant layer (modified OHW theory)
ζ_ELS2_^d+s^	zeta potential calculated according to eqs 22–28 from ELS data for *Du*^d+s^, reflecting surface conductivity in both diffuse and stagnant layer (full DS theory)
κ*a*_CVI_	product of Debye–Hückel parameter κ (calculated on basis of eq 8 and the conductivity of the dispersion medium *K*_m_) and particle radius *a*; used for the interpretation of CVI data (for details refer to ref (68))
κ*a*_ELS_	product of Debye–Hückel parameter κ (calculated on basis of eq 7 and the electrolyte concentration of the dispersion medium) and particle radius *a*; used for the interpretation of ELS data (for details refer to ref (68))

a The superscript indicates the considered contributions of surface conductivity in the diffuse layer (d) and the stagnant layer (s). The subscript refers to the measurement method used, i.e., CVI for colloid vibration current data, ELS for electrophoretic light scattering data.

### Estimation of κ*a* Values

In this study, we used two different dilution protocols based on the available sample quantities. In the case of equilibrium dilution, the stock dispersion was diluted with equilibrium supernatant extracted by centrifugation. This method ensured that the composition and the ionic strength of the dispersion medium did not change during the dilution process. Accordingly, the structure of the EDL was preserved and all related parameters remained constant.

In the case of nonequilibrium dilution, we added electrolyte solution with composition that might deviate from the composition of the original dispersion. We tried to match both compositions, but some deviations still remained. If the two compositions differ significantly, certain impacts on the ionic strength and electrokinetic parameters are to be expected. Information regarding the effects of the dilution can be obtained from the conductivity measurements of the dispersion medium. These are reported and discussed in detail in our recent publication.^68^ For a better understanding of the results in this study, we classify the various dispersion samples in terms of their dilution behavior using the κ*a* values.

The electrolyte composition is not known for the CVI measurements, as the original dispersant was not analyzed. We have therefore calculated κ using eq 8 and the conductivity of the dispersion medium *K*_m_. The data at various particle volume fractions were averaged and are shown in Table 3 in the column labeled κ*a*_CVI_. In the case of ELS measurements, the samples were diluted to such an extent that eq 7 with the target concentration listed in Table 1 can be used for κ determination. The corresponding κ*a* values are presented in Table 3 in the column labeled κ*a*_ELS_. However, the PVC samples were diluted with their supernatant, so that the κ*a*_CVI_ values were adopted in these cases.

**Table 3:** Compilation of κ*a* Values, Zeta Potentials ζ, and Dukhin Numbers *Du*^a^

material	κ*a*_CVI_^b^	κ*a*_ELS_^b^	ζ_CVI_^d+s^ in mV	ζ_ELS_^d^ in mV	ζ_ELS1_^d+s^ in mV	ζ_ELS2_^d+s^ in mV	*Du*^d+s^^b^	*Du*^d^	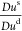
PVC@0.20	36.3 ± 0.3	36.3 ± 0.3	–87.1 ± 1.5	–83.2 ± 11.4	–102.2 ± 14.0	–102.5 ± 14.0	0.410	0.152	1.70
PVC@0.78	216.5 ± 9.4	216.5 ± 9.4	–84.4 ± 3.3	–73.6 ± 6.8	–93.7 ± 8.7	–93.3 ± 8.6	0.254	0.024	9.58
PVC@2.09	234.5 ± 2.9	234.5 ± 2.9	–111.4 ± 3.6	–94.4 ± 4.4	–123.7 ± 5.7	–123.5 ± 5.7	0.283	0.044	5.43
SBR@0.23	34.3 ± 1.7	38.3	–97.5 ± 2.6	–95.8 ± 2.9	–102.1 ± 3.0	–100.7 ± 3.0	0.256	0.214	0.20

a The subscript refers to the measurement technique used (the number in the subscript indicates solutions obtained using different evaluation models: 1 refers to the modified OHW theory and 2 to the DS theory) and the superscript to the surface conductivity contribution considered. *Du*^d+s^ is determined experimentally on the basis of MWO theory and covers both the contribution of the diffuse layer (*Du*^d^) and that of the stagnant layer (*Du*^s^). *Du*^d^ is calculated according to eq 9 on the basis of the ζ_CVI_^d+s^ values and *Du*^s^ as the difference of *Du*^d+s^ and *Du*^d^. Calculated standard deviations are absolute values from triplicate measurements.

b Values determined in our recent publication. For further details, refer to ref (68).

All PVC samples show genuine equilibrium dilution, indicated by the low standard deviations of the κ*a*_CVI_ values (<5%). These samples cover a broad spectrum of particle sizes, ranging from 0.20 to 2.09 μm. In particular, PVC@0.78 and PVC@2.09 have a very thin double layer, as κ*a* values are greater than 200.

The SBR@0.23 sample also has a relatively low standard deviation for the κ*a*_CVI_ value (approximately 5%), which is almost comparable to those of the PVC samples. Moreover, it agrees very well with the κ*a*_ELS_ value. These findings indicate only minor changes in the ionic strength within the dilution series and are also a hint for the reliability of the approximation eq 8. Thus, a well-defined electrolyte concentration can be set and maintained within the dilution series after the original electrolyte background has been eliminated by dialysis, meaning that almost equilibrium dilution is achieved.

In contrast, the samples PU@0.07 and PBAMM@0.10 show clear signs of nonequilibrium dilution, as indicated by the relatively high standard deviation of the κ*a*_CVI_ values (>15%). Since this also affected the quality of the further investigations, their results are only listed and discussed in the Supporting Information (Section 4).

### Zeta Potential Determination and Dukhin Number

In this study, we use CVI and ELS measurements to determine the zeta potential of polymer dispersions. In a previous publication, we analyzed the data within the framework of the Helmholtz–Smoluchowski and Henry model.^68^ In this section, we now present the results obtained with more advanced models that consider the relaxation effect and surface conductivity. The characterization of particle surface conductivity is carried out by means of the Dukhin number, which relates the surface conductivity to that of the dispersion medium. We determined this parameter on the basis of electrical conductivity data using the MWO theory. In addition, we also applied the Bikerman theory to carry out a more detailed analysis of the individual contributions to the surface conductivity.

Since the method of sample dilution can influence electrokinetic properties, we focus here on the samples in which equilibrium dilution was present or almost achieved. This is especially true for all PVC dispersions and the styrene–butadiene rubber SBR@0.23. As already mentioned, the samples PU@0.07 and PBAMM@0.10 revealed noticeable effects of nonequilibrium dilution. This is also reflected in their electrokinetic results, which, however, do not show any fundamental differences or new findings compared to the other samples. For the sake of completeness and with regard to our previous publication, we have collated the results of these two samples in the Supporting Information (Section 4).

In the following two sections, the basic investigation approach is explained in detail using the sample PVC@0.20. We will then discuss the differences and peculiarities of the other samples.

#### Discussion of Sample PVC@0.20 for the CVI Method

**Figure 1:**
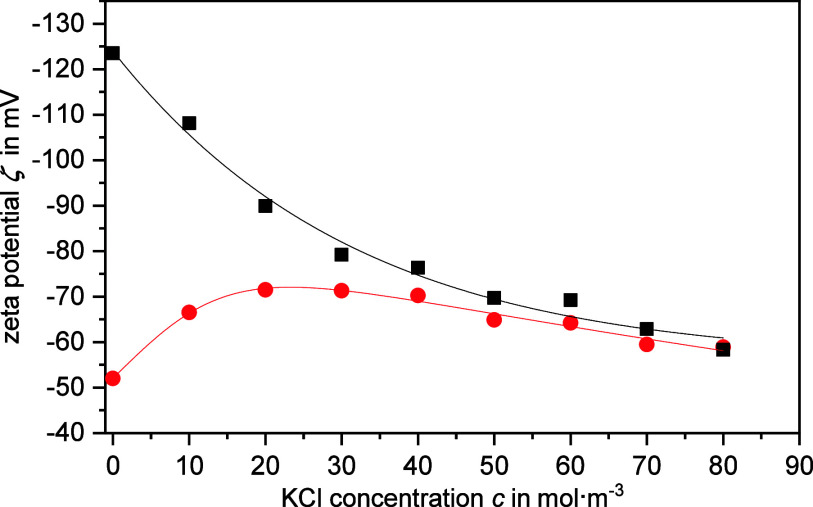
Zeta potential ζ determined with the CVI method as a function of KCl concentration *c* for sample PVC@0.20 at a particle volume fraction of approximately φ = 0.113. The zeta potentials are shown on the primary *y*-axis, where square refers to ζ using the advanced zeta potential theory and *Du*^d+s^ values and circle refers to ζ using the Henry theory. The pH values of the samples were 8.2. Solid lines are a guide to the eye.

In accordance with the IUPAC recommendations,^12^ we have investigated the dependence of the zeta potential on the electrolyte (KCl) concentration using the sample PVC@0.20 as an example. Among others, the measured CVI signals were analyzed using the Henry theory, as described in our previous publication.^68^ The results are shown in Figure 1 (circle) and listed in the Supporting Information (Section 2.2).

Figure 1 clearly shows a pronounced extremum at an electrolyte concentration of approximately 20 mmol/L, indicating a significant impact of surface conductivity on the electrokinetic measurement. The zeta potential shows values of up to −71.5 mV at quite low electrolyte concentrations. Such high values in magnitude also suggest that a mere consideration of the retardation effect, as described by the Henry theory, is no longer sufficient for a reliable evaluation. Consequently, subsequent zeta potential calculations were conducted using the advanced CVI theory, which accounts for both the relaxation effect and the surface conductivity of the particles. However, this approach requires knowledge of the Dukhin number.

**Figure 2:**
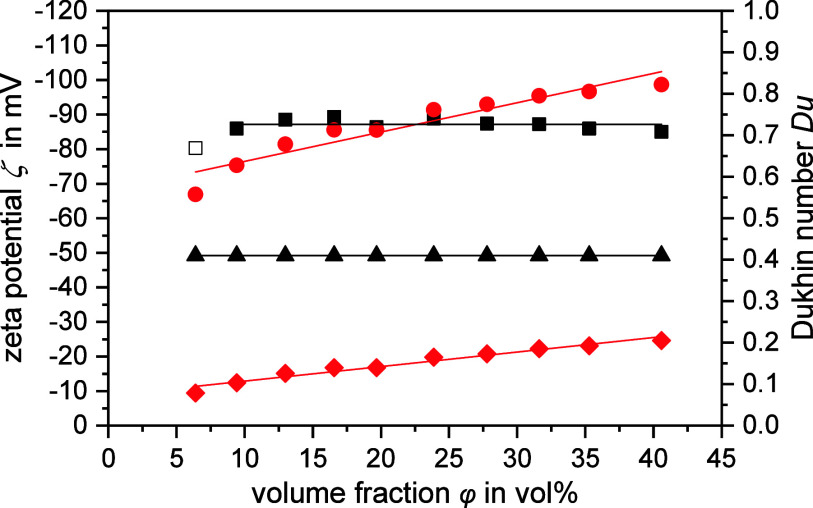
Zeta potential ζ determined with the CVI method as a function of particle volume fraction φ for sample PVC@0.20. The zeta potentials are shown on the primary *y*-axis, where square refers to ζ using Dukhin number *Du*^d+s^ and circle refers to ζ using *Du*_DT_^d^ values estimated by the CVI software on the basis of the measured dispersion conductivity. The corresponding Dukhin numbers are shown on the secondary *y*-axis, where diamond represents *Du*_DT_^d^ estimated by the CVI software and triangle *Du*^d+s^. Filled data points (square) were considered for the calculation of the average zeta potential value ζ_CVI_^d+s^ (shown as black solid line and listed in Table 3), whereas unfilled data points (open square) were neglected. The pH values of the samples were 9.0. The electrolyte concentrations were unknown, as the samples were diluted with their supernatant to maintain equilibrium dilution.

Figure 2 shows the zeta potentials and the corresponding Dukhin numbers as a function of the particle volume fraction. Similar plots can be found for all other dispersions investigated in the Supporting Information (Figure S1), and essential parameters are provided in Table 3.

The Dukhin number is mandatory for the calculation of the zeta potential based on the advanced CVI theory. The software of the CVI device used offers the option to calculate this value iteratively from the measured conductivity of the dispersion *K*_s_ with an approach following Bikerman’s theory. This means that these values only account for the contribution of surface conductivity within the diffuse layer, i.e., *Du*_DT_^d^. Figure 2 shows that the Dukhin numbers (diamond) determined in this way are relatively low and rise slightly with increasing volume fraction. On the other hand, the magnitude of the corresponding zeta potentials ζ_CVI_^d^ (circle) increases strongly from −67 to −99 mV. This behavior is unsatisfactory, as it contradicts the expectations for equilibrium dilution of the measured samples.

As an alternative, the Dukhin number can be determined experimentally, e.g., using the measured conductivity of the dispersion medium *K*_m_ and the dispersion *K*_s_. Several theories are known, which relates the relative conductivity *K*_s_ · *K*_m_^–1^ of a dispersion of nonconducting spherical particles to the Dukhin number and the volume fraction φ. We tested some of them to ensure that we were using a well-suited theory for our data analysis. The results are presented in Section 2.3 in the Supporting Information. It turned out that the evaluation with the MWO theory (cf. eq 11) provides a reliable Dukhin number (triangle in Figure 2). For clear distinction, we denote these values with *Du*^d+s^. This method gives a value of 0.410 for PVC@0.20, which is also the highest value of all dispersions studied. It was likewise found that *Du*^d+s^ is significantly higher than the *Du*_DT_^d^ values determined by the software. This is mainly due to the fact that the method based on MWO theory also considers SLC (i.e., *Du*^s^, calculated as the difference between *Du*^d+s^ and *Du*^d^ according to eq 3). Moreover, the contribution of SLC to the total surface conductivity of this dispersion is larger than that of DLC (*Du*^s^/*Du*^d^=1.70, where *Du*^d^ is calculated according to the Bikerman theory using eq 9 and the ζ_CVI_^d+s^ value). The software of the CVI device offers the option to enter the Dukhin number manually and to include it in the calculation of the zeta potential. Using the experimentally determined Dukhin number *Du*^d+s^, the resulting zeta potentials (square) are almost constant above a volume fraction of approximately 10 vol %. Hence, they fulfill an essential plausibility criterion, because the zeta potential is a unique characteristic of a charged interface under given conditions.^12^ Moreover, the corresponding mean value ζ_CVI_^d+s^ (−87.1 mV) is very reasonable, as a comparison with the results of the ELS method will show.

The particle volume fractions investigated in the CVI experiment are partly very high, so that the EDLs of individual particles might overlap. This could have an impact on the charge regulation in the interfacial area and consequently on the determined zeta potentials. In this case, the magnitude of the zeta potentials would rise according to a recent study of A. Dukhin and Reisel.^77^ Due to the uniform ζ_CVI_^d+s^ values obtained in our study for equilibrium dilution, the influence of EDL overlap is considered to be small.

The plausibility of the advanced CVI theory can also be tested by analyzing the zeta potentials measured at different electrolyte concentrations. As the diffuse layer is compressed with increasing ionic strength, the potential at the slip plane decreases. Therefore, the concentration-dependent extremum of the zeta potential should no longer be present if the effect of surface conductivity is properly taken into account. This is indeed observed, as shown in Figure 1 (square), where the absolute values of the zeta potentials decrease monotonically.

**Figure 3:**
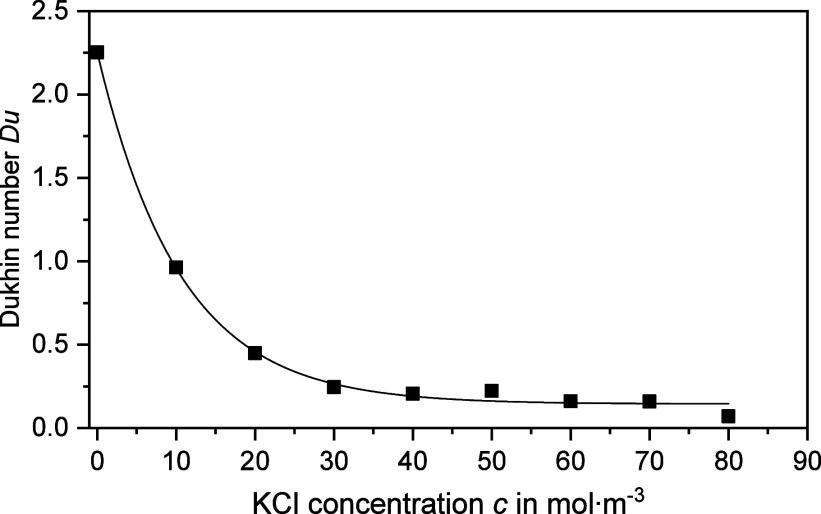
Dukhin number *Du*^d+s^ (square) as a function of electrolyte concentration *c* for sample PVC@0.20 at a particle volume fraction of approximately φ = 0.113. The Dukhin numbers were estimated from the relative conductivities *K*_s_ · *K*_m_^–1^ on the basis of eq 11 in the main article. The pH values of the samples were 8.2. The solid line is a guide to the eye.

The corresponding Dukhin numbers are presented in Figure 3. It was found that the determined *Du*^d+s^ values are well above 0.5 at low KCl concentrations. In other words, the dilution of the stock dispersion results in an overall electrolyte content below the isoconductive point, which has a major influence on the zeta potentials determined (see Figure 1). As the KCl concentration increases, the conductivity of the dispersion medium *K*_m_ also increases. Consequently, the Dukhin number decreases according to eq 1 and the influence of the surface conductivity *K*^σ^ on the zeta potential determination diminishes. The evaluations with the Henry and the advanced CVI theory therefore lead to comparable results at high KCl concentrations (see Figure 1).

#### Discussion of Sample PVC@0.20 for the ELS method

To check the plausibility of the CVI results, we followed Lyklema’s recommendation and performed an “electrokinetic consistency test”, i.e., the zeta potential was determined based on a different electrokinetic property in the same given dispersion medium.^78^ For this purpose, we employed the ELS method. The determined κ*a* value is 36.3. Thus, the condition of a thin diffuse layer is met, which is required when applying many models. Accordingly, the measured electrophoretic mobility can be evaluated using eqs 14–20 by Ohshima, Healy, and White. The value obtained for ζ_ELS_^d^ is −83.2 mV, which is nearly identical to ζ_CVI_^d+s^ at −87.1 mV. At first glance, this finding seems plausible, since Delgado et al. pointed out that SLC significantly affects electrokinetic studies only when *Du*^s^/*Du*^d^ exceeds 2–5.^12^ However, PVC@0.20 is close to this condition with a value of 1.70 and it should be noticed that the contribution of SLC to the surface conductivity is already considerable.

For comparison, we have also determined the zeta potential using a modification of the OHW theory, i.e., we have included eq 21 instead of eq 16 in the calculation. This allows the influence of the SLC to be taken into account in the OHW theory if the determined *Du*^d+s^ value is employed instead of *Du*^d^ for the Dukhin number. The value calculated in this way is −102.2 mV (cf. ζ_ELS1_^d+s^ in Table 3) and is somewhat higher in magnitude than the ζ_CVI_^d+s^ value. In order to recognize incorrect assumptions when modifying the OHW theory, it is useful to calculate the zeta potentials additionally by means of the DS theory, which has rarely been applied in the past.^47−56^ In the following, we will explain the main features of this approach in detail.

Equation 22 describes the relation between the electrophoretic mobility μ obtained from ELS measurement and the Stern and zeta potential. The parameter *p* is also required, which describes the ratio of the ion diffusion coefficient in the boundary layer to that in the bulk medium. The influence of these parameters on the Dukhin number is given by eq 28. The zeta potential was calculated in former studies on the basis of eq 22 and experimentally determined values of the electrophoretic mobility and the Stern potential. A plausible value of 0.85 for the parameter *p* was additionally adopted, as suggested by Moncho et al. for polystyrene particles.^55^ In a new alternative approach, we have now used the Dukhin number instead of the Stern potential. Based on the experimentally determined values for μ and *Du*^d+s^, it is possible to solve the system of eqs 22–28 numerically and obtain values for the Stern and zeta potential.

In principle, we obtain two solution sets: a plausible one with the correct sign for both potentials and an incorrect one, where the Stern potential has the opposite sign to the electrophoretic mobility and the zeta potential. In addition, the zeta potentials in both solutions differ considerably. The correct zeta potential is listed in Table 3 in the column labeled ζ_ELS2_^d+s^. For comparison, all other results can be found in the Supporting Information in Table S4.

Before we take a closer look at the individual results, it makes sense to determine the influence of the approximately estimated parameter *p* and take it into account in the discussion. For this purpose, we varied this parameter in a range from 0.55 to 1.0. The associated zeta and Stern potentials are also listed in the Supporting Information in Table S5. We found that the values for the Stern potential ψ_d_ lie within a range of −135.5 to −152.2 mV. Obviously, the variation of the parameter *p* has a distinct influence on the Stern potential, meaning that an exact value cannot be reliably determined in our study. The differences, however, are so small that at least the order of magnitude of the Stern potential can be estimated. Remarkably, the zeta potential remains practically unaffected by the variation of the parameter *p*, as we found a uniform value −102.5 mV. This finding is of great importance as it allows the determination of the zeta potential even without precise knowledge of the parameter *p*.

Equation 29 is a simplified approximation of eq 22, with the advantage that the parameter *p* no longer needs to be known.^45,46^ To check the accuracy and reliability of this straightforward formula, we also calculated the zeta potential using the experimentally determined Dukhin number *Du*^d+s^. The Stern potential can subsequently be determined by means of eq 28, again assuming *p* = 0.85. In this way, we obtained a value of −100.2 mV for the zeta potential and a value of −140.4 mV for the Stern potential (cf. Table S4 in the Supporting Information). These values are in excellent agreement with the results of eq 22. This finding is also confirmed for the other polymer samples investigated in this study. It can therefore be concluded that eq 29 offers a very good and simple alternative to eq 22. However, in the case of negative electrophoretic mobilities, it is essential for eq 29 to divide by the absolute value of the dimensionless zeta potential. Otherwise, the second incorrect value for the zeta potential is calculated, as determined when solving eq 22. The excellent agreement of the results obtained with eqs 22 and 29 also proves that the knowledge of the parameter *p* is not necessary for the determination of the zeta potential if the Dukhin number is determined experimentally.

The close agreement between ζ_ELS1_^d+s^ (−102.2 mV) and ζ_ELS2_^d+s^ (−102.5 mV) underscores the reliability of both approximation formula. These values are higher in magnitude than the ζ_ELS_^d^ value (−83.2 mV), as was to be expected due to the contribution of the SLC to the surface conductivity. In this context, the comparison with the CVI method is also of particular interest. The ζ_CVI_^d+s^ value (−87.1 mV) is only slightly higher in magnitude than the ζ_ELS_^d^ value. Although the differences to ζ_ELS1_^d+s^ and ζ_ELS2_^d+s^ is noticeable, it should be recognized that all these methods adequately account for the contribution of the SLC. Thus, they provide more reliable zeta potentials than the original OHW approach if SLC is significant.

Our results reveal that PVC@0.20 contains highly charged particles with high surface potential. For this reason, relaxation effects are likely to occur. Consequently, the magnitude of the zeta potential is significantly higher than the value calculated according to the Henry theory (see Table S4 in the Supporting Information) if the surface conductivity is taken into account correctly.

#### Discussion of Samples PVC@0.78 and PVC@2.09

**Figure 4:**
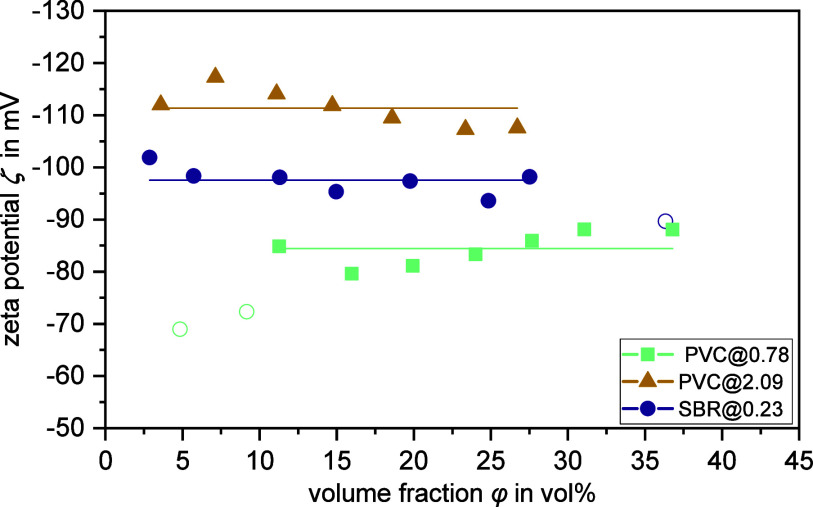
Zeta potential ζ measured by the CVI method as a function of particle volume fraction φ for samples PVC@0.78, PVC@2.09, and SBR@0.23. The Dukhin number *Du*^d+s^ determined experimentally on the basis of the MWO theory was used. Filled data points were considered for the calculation of the average zeta potential value ζ_CVI_^d+s^ (shown as solid line and listed in Table 3), whereas unfilled data points were neglected. The pH values of the samples were 6.8 for PVC@0.78, 9.6 for PVC@2.09, and 8.9 for SBR@0.23. The electrolyte concentrations of the PVC samples were unknown, as the samples were diluted with their supernatant to maintain equilibrium dilution. SBR@0.23 was first dialyzed and then diluted to achieve a KCl concentration of 10 mmol L^–1^ in the dispersion medium.

The comparison of the results from PVC@2.09 and PVC@0.78 reveals some similarities (see Table 3 and Figure 4). In contrast to PVC@0.20, these samples are characterized by large particle sizes with very thin diffuse layers (κ*a* > 200). The *Du*^d+s^ values show that the effect of the surface conductivity in relation the dispersion conductivity is not as pronounced as for PVC@0.20. However, it should be noticed that the SLC contribution to the total surface conductivity is dominant for PVC@0.78 and PVC@2.09, with PVC@0.78 having the highest value of all dispersions studied (*Du*^s^/*Du*^d^ = 9.58).

As already explained for PVC@0.20, the high SLC contribution causes distinct differences in the various zeta potentials, listed in Table 3. Again, reliable zeta potentials can only be determined if both contributions of the surface conductivity are correctly taken into account by the Dukhin number. The use of *Du*^d+s^ in the CVI method leads to consistent zeta potentials over a wide volume fraction range (cf. Figure 4). The corresponding average value ζ_CVI_^d+s^ again matches the values from the modified OHW and DS theory quite well but always tends to be lower in magnitude. On the other hand, the values for ζ_ELS1_^d+s^ and ζ_ELS2_^d+s^ show excellent agreement with only negligible differences. It is particularly important to note that neglecting the contribution of SLC in the original OHW theory leads to ζ_ELS_^d^ values, which are considerably lower in magnitude. In this respect, it can again be stated that the consideration of the *Du*^d+s^ values in the other theories significantly increases the reliability of the determined zeta potential.

#### Discussion of Sample SBR@0.23

As explained in Estimation of κa Values, the κ*a* values of SBR@0.23 changed only slightly upon dilution, suggesting that almost equilibrium dilution was achieved. This is also reflected in the zeta potentials measured with the CVI method as a function of the particle volume fraction. Figure 4 shows that SBR@0.23 exhibits consistent zeta potentials with only minor fluctuations over a wide range of particle concentrations.

It was found that the effect of surface conductivity is also relevant for SBR@0.23 (*Du*^d+s^ = 0.256). However, the analysis of the Dukhin numbers indicates that the contribution of the SLC can be neglected (*Du*^s^/*Du*^d^ = 0.20). The major contribution of the DLC is also reflected in the determined zeta potentials, as ζ_ELS_^d^ (−95.8 mV) and ζ_CVI_^d+s^ (−97.5 mV) are in excellent agreement, with only ζ_ELS1_^d+s^ (−102.1 mV) and ζ_ELS2_^d+s^ (−100.7 mV) being slightly higher in magnitude. This is an important result because ζ_ELS_^d^ is based on the original OHW theory,^43^ which is regarded as a reliable alternative to the generally accepted reference theory of O’Brien and White^75^ for the case of negligible SLC. Similar observations were reported for polystyrene particles when the ELS method was used in combination with the standard electrokinetic model assuming a constant surface charge density and DLC only. It was shown that the calculated zeta potentials reliably describe the measured electrophoretic mobilities even at very low electrolyte concentrations.^79,80^

Consequently, it can be concluded that not only the modified OHW theory but also the advanced CVI theory and the DS theory correctly account for the DLC when the thickness of the diffuse layer is sufficiently thin. This in turn implies that the differences observed for the PVC samples between ζ_CVI_^d+s^ on the one hand and ζ_ELS1_^d+s^ and ζ_ELS2_^d+s^ on the other hand are probably due to a slightly different consideration of SLC in the underlying theories. Based on our results, however, it is hardly possible to decide which values are more accurate. In this respect, further investigations using alternative electrokinetic methods would be helpful, as they could provide additional information.

## Conclusions

Zeta potentials of polymer dispersions were determined for a wide range of solid content by electroacoustic measurement of the CVI. They were compared with values determined by ELS in diluted dispersion in order to perform an “electrokinetic consistency test”.^78^ When evaluating the primary ELS measurement results, we used the full and simplified versions of the Dukhin–Semenikhin (DS) theory^44−46^ as well as the original and modified versions of the Ohshima–Healy–White (OHW)^43^ theory. Our modification now allows the Dukhin number to be taken into account. The advanced CVI theory^2,70^ was applied to the CVI data, which particularly enables the measurement of dispersions with high volume fractions. In a previous publication,^70^ the dependence on volume fraction has already been validated. In this study, we verify for the first time the proper consideration of surface conductivity in the advanced CVI theory. We also pursue a new approach that combines conductivity measurements of the dispersion and dispersion medium with the electroacoustic and electrophoretic zeta potential determination. Moreover, a detailed analysis of the conductivity within the stagnant layer (SLC) and in the diffuse layer (DLC) is given, as both can play an important role for the surface conductivity.

The Dukhin number was used as a measure for the contribution of SLC and DLC to electrokinetic and electroacoustic effects. The experimental determination was carried out by measuring the conductivities of the dispersion and the dispersion medium at different particle volume fractions, as described in a recent publication.^68^ Preferably, the solid content of the dispersions should be adjusted by equilibrium dilution with supernatant in order to avoid effects on the properties of the EDL. The evaluation of these data was based on the Maxwell–Wagner–O’Konski (MWO) theory.^36−38^ As a result, the Dukhin number *Du*^d+s^ is obtained, which reflects the contribution of both the stagnant (*Du*^s^) and the diffuse layer (*Du*^d^) to the surface conductivity. The contribution of the diffuse layer alone can be estimated by means of the Bikerman theory.^12^ Comparison of these two values of Dukhin number yields information on the surface conductivity in the stagnant layer.

Our study reveals that the surface conductivity is of significant importance for all investigated polymer dispersions. It has been found that this effect must be correctly taken into account in the CVI measurement in order to determine zeta potential values that are not influenced by the volume fraction. Furthermore, the analysis of the Dukhin numbers proved that in many cases, not only the DLC but also the SLC contributes considerably to the surface conductivity. Neglecting the SLC contribution can result in zeta potential values whose magnitudes are considerably lower than the correct values.

We have demonstrated for the first time that reliable zeta potential values can be obtained for a wide volume fraction range using the CVI method in combination with the advanced CVI theory. These values agree well with those obtained by the ELS method. As expected, significant deviations to the original OHW theory are only observed for samples with a much higher conductivity in the stagnant than in the diffuse layer, since this proven theory does not account for SLC. This limitation can be overcome by employing the rarely used DS theory or the modified OHW theory for analyzing the ELS data. These approaches provide reasonable zeta potentials with values that are only slightly higher in magnitude than those obtained with the advanced CVI theory.

Moreover, our study reveals that the knowledge of the parameter *p*, which accounts for differences in the ion diffusion coefficients in the stagnant layer compared to the bulk medium, is not a prerequisite for the zeta potential determination when using the full DS theory. However, this requires knowledge of the Dukhin number as an essential input parameter. We also find an excellent agreement between the full and the simplified version of the DS theory. This is a very useful result as it shows the possibility of simplifying the calculation procedure for the zeta potential.

In summary, these results highlight the benefits of conductivity measurements of the dispersion and the dispersion medium for electrokinetic investigations. The common link between these methods is the Dukhin number *Du*^d+s^, which can be determined experimentally in a simple and reliable way. This parameter allows the influence of both the SLC and the DLC to be correctly taken into account when determining the zeta potential.

## Data Availability

Data that support the findings of this study are available from the corresponding author upon reasonable request.

## Supporting Information Available

Particle size distributions; Dukhin numbers and zeta potentials determined using the advanced CVI theory; Dukhin numbers determined using alternative theories; additional results obtained with the Dukhin–Semenikhin theory; results of samples subjected to nonequilibrium dilution (PDF)
