# Supplementary material for: New Experimental Approach for the Proper Consideration of Stagnant and Diffuse Layer Conductivity in the Zeta Potential Determination
Source: Langmuir. 2025 Feb 19;41(8):5188–201. doi: 10.1021/acs.langmuir.4c04456 (PMC11887656; doi:10.1021/acs.langmuir.4c04456)
Supplement: Supplementary file 1 — la4c04456_si_001.pdf [file la4c04456_si_001.pdf]

Supporting Information

New Experimental Approach for the Proper Consideration of Stagnant and Diffuse Layer Conductivity in the Zeta Potential Determination

Matthias Frangenberg[a,b], Annette M. Schmidt[b], Jan Wilkens[a,*]

[a] Faculty of Applied Natural Sciences, TH Köln – University of Applied Sciences, D-51379 Leverkusen, Germany
[b] Department of Chemistry, Institute of Physical Chemistry, University of Cologne, D-50939 Cologne, Germany
* E-Mail: jan.wilkens@th-koeln.de; Phone: +49 214-32831-4614; Address: Campusplatz 1, D-51379 Leverkusen, Germany

Table of Contents

1. Particle Size Distribution of the Investigated Samples S2
2. Dukhin Numbers and Zeta Potentials Determined Using the Advanced CVI Theory S2
2.1. Dependency on Volume Fraction S2
2.2. Dependency on Electrolyte Concentration S3
2.3. Determination of the Dukhin Number using Alternative Theories S4
3. Compilation of Results Obtained with the Henry and Dukhin-Semenikhin Theory S5
3.1. Zeta and Stern Potentials determined using the Henry and Dukhin-Semenikhin Theory S5
3.2. Influence of the Parameter p on the Determination of the Zeta and Stern Potential S6
4. Results and Discussion of the Samples PU@0.07 and PBAMM@0.10 .... S7
4.1. Estimation of κa Values S7
4.2. Zeta Potential Determination and Dukhin Number S7
5. References S8

Number of pages: 8

Number of figures: 4

Number of tables: 6

1. Particle Size Distribution of the Investigated Samples

The particle size distributions (PSD) of most samples were determined using dynamic light scattering at 25°C with a ZetaSizer Nano ZS from Malvern Panalytical (633 nm wavelength and backscatter detection at 173°). Measurements were conducted in poly(methyl methacrylate) macro cuvettes, with each measurement comprising 15 sub-runs. Samples were diluted according to the protocol described in section 3.2.2 of the main article. They were measured at different particle volume fractions to ensure that multiple light scattering is discovered. The PSDs of samples PVC@0.78 and PVC@2.09 were determined using laser diffraction spectroscopy with a MasterSizer 2000 (633 nm and 433 nm wavelength) from Malvern Panalytical. The measurements were carried out in triplicate. Median size and standard deviation are the necessary input parameters of a lognormal particle size distribution in the DT-1202 CVI software. More details can be found in our recent publication [1].

Table S1: Characteristic data for the volume-weighted particle size distribution

sample	d_{10} in µm	d_{50} in µm	d_{90} in µm	d_{mean} in µm	standard deviation
PVC@0.20	0.129	0.202	0.314	0.212	0.160
PVC@0.78	0.354	0.783	1.377	0.834	0.136
PVC@2.09	1.057	2.086	4.223	2.416	0.255
SBR@0.23	0.063	0.233	0.575	0.270	0.327
PU@0.07	0.045	0.069	0.127	0.079	0.220
PBAMM@0.10	0.069	0.099	0.155	0.105	0.163

2. Dukhin Numbers and Zeta Potentials Determined Using the Advanced CVI Theory

2.1. Dependency on Volume Fraction

Individual Dukhin numbers and the corresponding zeta potentials determined with the CVI measuring method are compared in Fig. S1 as a function of volume fraction φ. On the one hand, the particle surface conductivity is characterized by the experimentally determined Dukhin number $Du^{\mathrm{d+s}}$, which is based on the Maxwell-Wagner-O'Konski (MWO) theory and reflects surface conductivity in both diffuse and stagnant layer. On the other hand, the approach of the CVI software is used, which iteratively determines the Dukhin number $Du_{\mathrm{DT}}^{\mathrm{d}}$ on the basis of the measured dispersion conductivity K_{s} following the Bikerman theory. Therefore, it reflects surface conductivity in the diffuse layer only. The comparison contains all samples that are not included in the main article.

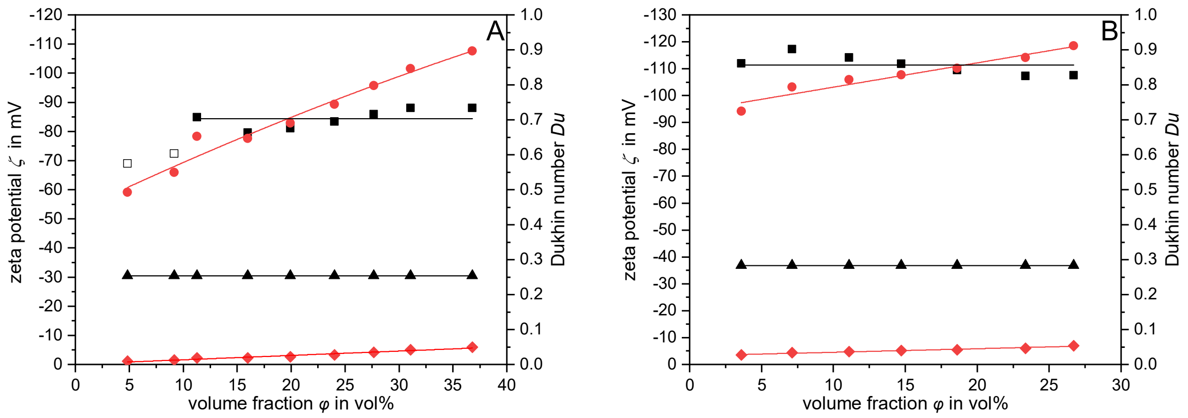

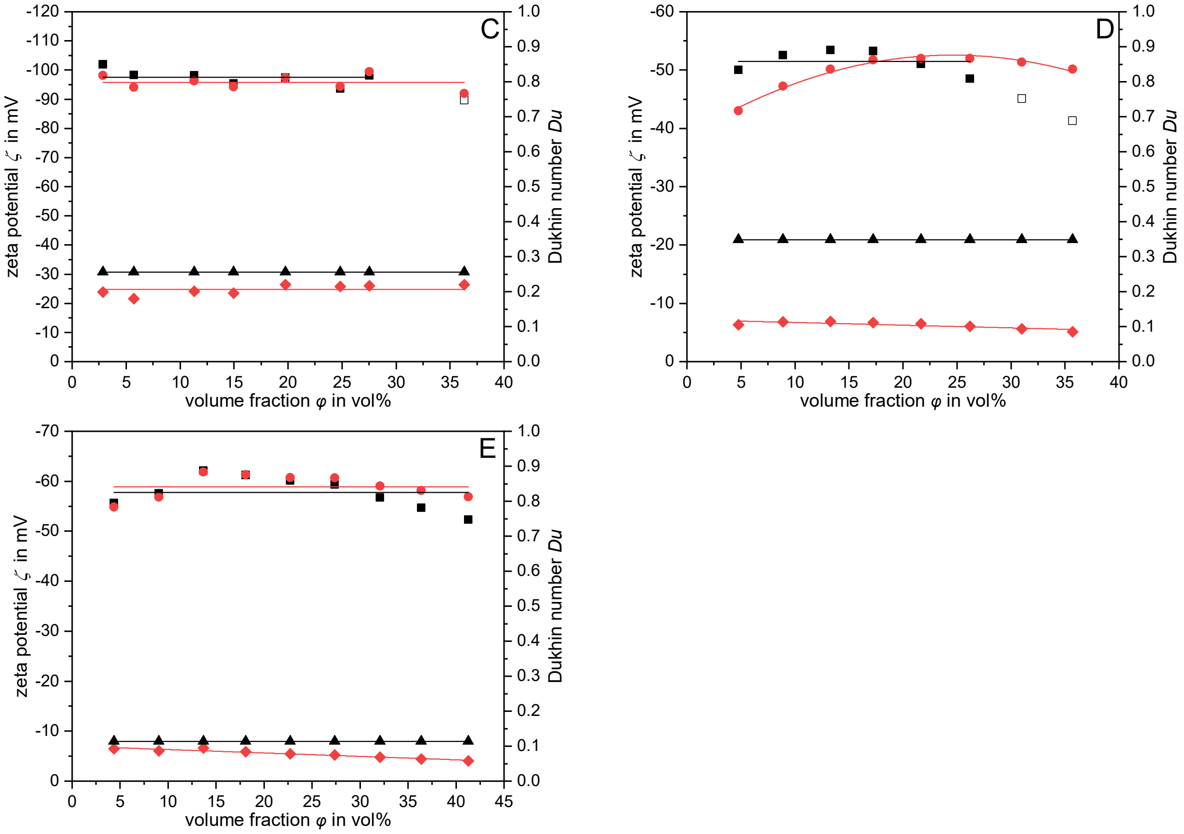

Figure S1. Zeta potential ζ determined with the CVI method as a function of particle volume fraction φ, where A is PVC@0.78, B is PVC@2.09, C is SBR@0.23, D is PU@0.07 and E is PBAMM@0.10. The zeta potentials are shown on the primary y-axis, where ■ refers to ζ using Dukhin number $Du^{\mathrm{d+s}}$ and ● refers to ζ using $Du^{\mathrm{d}}_{\mathrm{DT}}$ values estimated by the CVI software on the basis of the measured dispersion conductivity. The corresponding Dukhin numbers are shown on the secondary y-axis, where ◆ represents $Du^{\mathrm{d}}_{\mathrm{DT}}$ estimated by the CVI software and ▲ $Du^{\mathrm{d+s}}$. Filled data points (■) were considered for the calculation of the average zeta potential value $\zeta^{\mathrm{d+s}}_{\mathrm{CVI}}$ (shown as black solid line and listed in Table 3 of the main article and Table S6), whereas unfilled data points (□) were neglected.

2.2. Dependency on Electrolyte Concentration

We have investigated the dependence of the zeta potential on the electrolyte concentration using the sample PVC@0.20 and the CVI method. The stock dispersion was diluted with KCl solutions of varying concentrations to a particle volume fraction φ of approx. 11.3 vol %. All relevant data and results are summarised in Table S2.

Table S2: Dependence of the zeta potential ζ on the KCl concentration c for PVC@0.20 [a)]

c in mmol/L	κa	K_{m} in S/m	$Du^{\mathrm{d+s}}$	$\zeta_{\mathrm{CVI,H}}$ in mV	$\zeta^{\mathrm{d+s}}_{\mathrm{CVI}}$ in mV
0	17.1	0.0397	2.230	-52.0	-123.5
10	33.5	0.1520	0.960	-66.5	-108.1
20	43.2	0.2533	0.449	-71.5	-89.9
30	53.8	0.3930	0.245	-71.3	-79.2
40	60.1	0.4900	0.206	-70.2	-76.4
50	66.3	0.5963	0.224	-64.9	-69.7
60	73.2	0.7267	0.161	-64.2	-69.2
70	78.4	0.8337	0.160	-59.5	-62.9
80	83.9	0.9550	0.071	-58.9	-58.3

[a] Measurement of the electrical conductivity of the dispersion medium (K_m) is required for calculating the Debye-Hückel parameter κ using equation 8. The Dukhin number Du^{d+s} is determined using equation 11. The subscript of the zeta potentials refers to the measurement technique used for the determination of the electrophoretic mobility μ (CVI: colloid vibration current) and the superscript to the surface conductivity contribution considered (d+s: surface conductivity in both diffuse and stagnant layer). The subscript H indicates zeta potentials calculated according to the Henry theory, thus neglecting surface conductivity. The pH values of the samples were approx. 8.2.

2.3. Determination of the Dukhin Number using Alternative Theories

Accurate determination of the zeta potential in colloidal dispersions often requires consideration of the relaxation effect, which is associated with the polarization of the electrical double layer and the surface conductivity. In this study, we have investigated the particle surface conductivity by means of the Dukhin number Du, which is a measure for the significance of the surface conductivity K^{σ} compared to the medium conductivity K_m. This parameter is also mandatory for the zeta potential determination using the advanced CVI, the Dukhin-Semenikhin and a modification of the Ohshima-Healy-White theory to account for the influence of the surface conductivity.

The Dukhin number can be determined experimentally, e.g. by measuring the conductivity of the dispersion medium K_m and the dispersion K_s at different particle volume fractions φ. Several theories are known, which relate the relative conductivity $K_s \cdot K_m^{-1}$ of a dispersion of non-conducting spherical particles to the Dukhin number and the volume fraction. A good overview can be found in the publication by Van der Put and Bijsterbosch [2]. We tested the Henry-Booth or Bruggeman theory (HB-B; eq S1) and the Dukhin-Semenikhin theory (DS; eq S2). The obtained Dukhin numbers were compared with those resulting from the Maxwell-Wagner-O'Konski theory (MWO; eq 11 in the main article). As described in section 3.3 of the main article, the Dukhin number can be computed by least-squares fit using the cited equations. The results are shown in Table S3.

$$\left(\frac{K_s}{K_m}\right)^{\frac{1}{3}} = \frac{1}{1-\varphi} \cdot \left[\frac{\frac{K_s}{K_m} - 2 \cdot Du_{HB-B}^{d+s}}{1 - 2 \cdot Du_{HB-B}^{d+s}}\right] \tag{S1}$$

$$\left(\frac{K_s}{K_m}\right)^{\frac{4}{3}} = \frac{1}{1-\varphi} \cdot \left[\frac{\frac{K_s}{K_m} - Du_{DS}^{d+s}}{1 - Du_{DS}^{d+s}}\right]^2 \tag{S2}$$

The least squares fits yielded very good results for all theories and do not indicate a preference for one theory. However, considerable discrepancies can be observed between the Dukhin numbers determined. While the Du values of the MWO and the HB-B theory are quite similar, there are major differences to the values of the DS theory. Since PVC@0.20 has the largest Dukhin number of all dispersion studied, we selected this sample to investigate the influence of the different Dukhin numbers on the zeta potential determination.

Table S3: Dukhin number calculated on the basis of various theories [a]

material	Maxwell-Wagner-O'Konski Du_{MWO}^{d+s}	Henry-Booth and Bruggeman Du_{HB-B}^{d+s}	Dukhin-Semenikhin Du_{DS}^{d+s}
PVC@0.20	0.410	0.390	0.662
PVC@0.78	0.254	0.251	0.349
PVC@2.09	0.283	0.251	0.345
SBR@0.23	0.256	0.255	0.346
PU@0.07	0.348	0.329	0.512
PBAMM@0.10	0.114	0.107	0.136

[a] Du was determined by least-squares fit of the relative conductivity $K_s \cdot K_m^{-1}$ in dependence of the volume fraction φ using the above mentioned equations. The $K_s \cdot K_m^{-1}$ values were determined in our recent publication. For further details, refer to [1].

Zeta potentials of sample PVC@0.20 were determined with the CVI measuring method and are shown in Fig. S2 as a function of the volume fraction φ. Obviously, the magnitude of the zeta potentials, which are determined with the Dukhin number Du_{DS}^{d+s} according to the Dukhin-Semenikhin theory (●), decreases significantly from -96.5 mV to -78.3 mV with increasing volume fraction. This behaviour is unsatisfactory, as it contradicts the expectations for

equilibrium dilution of the measured samples. In contrast, the zeta potentials, which are calculated with the Dukhin number $Du_{\mathrm{MWO}}^{\mathrm{d+s}}$ according to the Maxwell-Wagner-O'Konski theory (■), are almost constant above a volume fraction of approximately 10 vol%. The same applies to the Dukhin number $Du_{\mathrm{HB-B}}^{\mathrm{d+s}}$, which is based on the Henry-Booth or Bruggeman theory (▲). Hence, both theories fulfil an essential plausibility criterion, because the zeta potential is a unique characteristic of a charged interface under given conditions [3].

The comparison of the zeta potentials as a function of the volume fraction clearly shows that the Dukhin numbers determined by means of both the MWO and the HB-B theory lead to very plausible results, especially compared to the DS theory. Due to the similarity of the results, there is no preference for one of the two theories. We therefore decided to follow the recommendations of International Standard ISO 13099, Part 1, and analysed the relative conductivities in this study using the MWO theory [4].

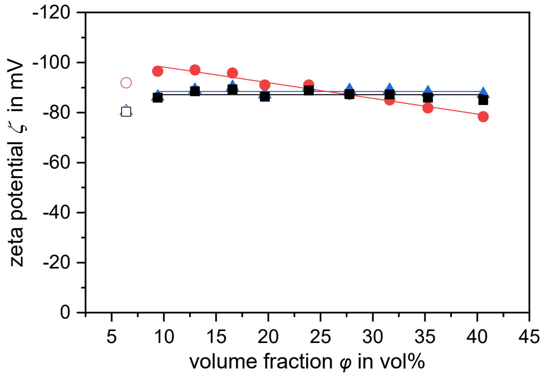

Figure S2. Zeta potential ζ determined with the CVI method as a function of particle volume fraction φ for sample PVC@0.20, where ■ refers to ζ using Dukhin number $Du_{\mathrm{MWO}}^{\mathrm{d+s}} = 0.410$, ▲ refers to ζ using Dukhin number $Du_{\mathrm{HB-B}}^{\mathrm{d+s}} = 0.390$ and ● refers to ζ using Dukhin number $Du_{\mathrm{DS}}^{\mathrm{d+s}} = 0.662$. The pH values of the samples were 9.0. The electrolyte concentrations were unknown, as the samples were diluted with their supernatant to maintain equilibrium dilution. Solid lines in the case of $Du_{\mathrm{MWO}}^{\mathrm{d+s}}$ and $Du_{\mathrm{HB-B}}^{\mathrm{d+s}}$ represent the average zeta potential values. The solid line in case of $Du_{\mathrm{DS}}^{\mathrm{d+s}}$ corresponds to a linear fit and is only a guide to the eye. Filled data points were considered for the calculation of the solid lines, whereas unfilled data points were neglected.

3. Compilation of Results Obtained with the Henry and Dukhin-Semenikhin Theory

3.1. Zeta and Stern Potentials determined using the Henry and Dukhin-Semenikhin Theory

The determination of the zeta and the Stern potential using the Dukhin-Semenikhin theory (DS theory) is described in detail in the main article. In this section, we essentially present the other, alternative results.

Table S4: Comparison of different zeta potentials ζ and Stern potentials ψ_{d} [a)]

material	μ in $\frac{\mu\mathrm{m \cdot cm}}{\mathrm{V \cdot s}}$	$\zeta_{\mathrm{CVI,H}}$ [b)] in mV	$\zeta_{\mathrm{ELS,H}}$ [b)] in mV	$\zeta_{\mathrm{ELS2}}^{\mathrm{d+s}}$ [c)] in mV	$\psi_{\mathrm{d,ELS2}}^{\mathrm{d+s}}$ [c)] in mV	$\zeta_{\mathrm{ELS3}}^{\mathrm{d+s}}$ [d)] in mV	$\psi_{\mathrm{d,ELS3}}^{\mathrm{d+s}}$ [d)] in mV	$\zeta_{\mathrm{ELS4}}^{\mathrm{d+s}}$ [e)] in mV	$\psi_{\mathrm{d,ELS4}}^{\mathrm{d+s}}$ [e)] in mV
PVC@0.20	-5.320	-71.8	-72.3	-102.5	-139.6	-80.8	145.8	-100.2	-140.4
PVC@0.78	-5.554	-70.5	-71.6	-93.3	-213.1	-79.9	214.0	-93.0	-213.2
PVC@2.09	-7.024	-92.7	-90.5	-123.5	-220.4	-100.9	222.5	-123.1	-220.4
SBR@0.23	-5.871	-83.7	-79.5	-100.7	-114.7	-85.8	121.8	-98.9	-115.8
PU@0.07	-3.308	-44.7	-51.9	-59.3	-85.6	-50.3	89.0	-57.1	-86.5
PBAMM@0.10	-4.276	-56.5	-61.7	-63.2	-45.6	-59.4	49.8	-62.1	-46.8

[a)] The subscript refers to the measurement technique used for the determination of the electrophoretic mobility μ (CVI: colloid vibration current; ELS: electrophoretic light scattering; H indicates zeta potentials calculated according to Henry theory, neglecting surface conductivity) and the superscript to the surface conductivity contribution considered (d+s: surface conductivity in both diffuse and stagnant layer). The number in the subscript indicates different solutions obtained using the Dukhin-Semenikhin theory (DS theory).

[b)] Zeta potentials calculated according to the Henry theory, as determined in our recent publication. For further details, refer to [1].

[c)] Correct solution of the system of equations 22-28 (the zeta and Stern potentials have the same sign as the electrophoretic mobility).

[d)] Incorrect second solution of the system of equations 22-28 (the zeta potential has the same sign as the electrophoretic mobility, whereas the Stern potential has the opposite sign).

[e)] Correct solution using the simplified equation 29 for the zeta potential. The Stern potential was subsequently calculated using equation 28.

It can be clearly seen that the values with index 2 (full DS theory) and 4 (simplified DS theory) correspond very well with each other. As explained in the main article, they represent plausible results. The values with the index 3 are the alternative solutions of the system of equations 22-28 (full DS theory). Since the Stern potentials have the opposite sign to the measured electrophoretic mobilities, these solution sets must be regarded as implausible.

3.2. Influence of the Parameter p on the Determination of the Zeta and Stern Potential

The determination of the zeta and the Stern potential using the full Dukhin-Semenikhin theory (DS theory) is described in detail in the main article. In this section, we present the results for PVC@0.20 obtained by varying the parameter p.

Table S5: Zeta potentials ζ and Stern potentials ψ_{d} for PVC@0.20, determined using the DS theory [a)]

material	p	$\zeta^{\mathrm{d+s}}_{\mathrm{ELS5}}$ [b)]	$\psi^{\mathrm{d+s}}_{\mathrm{d,ELS5}}$ [b)]
PVC@0.20	0.55	-102.5	-152.2
PVC@0.20	0.70	-102.5	-144.9
PVC@0.20	0.85	-102.5	-139.6
PVC@0.20	1.00	-102.6	-135.5

[a)] The subscript refers to the measurement technique used for the determination of the electrophoretic mobility μ (ELS: electrophoretic light scattering) and the superscript to the surface conductivity contribution considered (d+s: surface conductivity in both diffuse and stagnant layer). The number in the subscript is used to distinguish it from the other results.

[b)] Correct solution of the system of equations 22-28 (the zeta and Stern potentials have the same sign as the electrophoretic mobility).

The data in Table S5 clearly show that the Stern potential is strongly influenced by the variation of the parameter p. The zeta potential, on the other hand, remains practically unaffected by this variation (deviations only occur in the first decimal place). This result is of great importance as it allows the determination of the zeta potential even without precise knowledge of the parameter p.

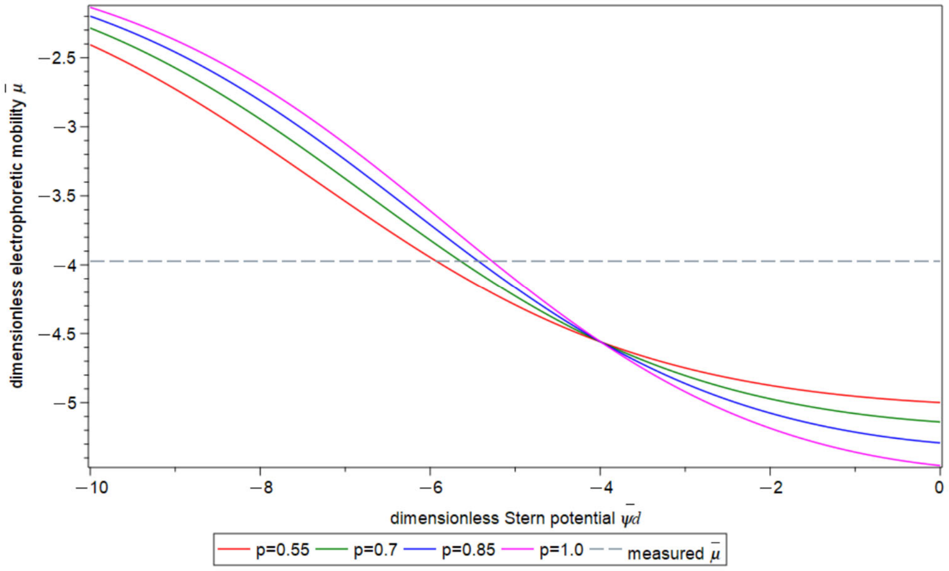

Figure S3. Plot of the dimensionless electrophoretic mobility $\bar{\mu}$ as a function of the dimensionless Stern potential $\overline{\psi_{\mathrm{d}}}$ for a given dimensionless zeta potential $\bar{\zeta}$ with a value of -3.991, which corresponds to $\zeta = -102.5$ mV. The parameter p is varied between 0.55 and 1.0. The associated, experimentally determined dimensionless electrophoretic mobility has a value of -3.973, which corresponds to $\mu = -5.320$ (µm·cm)/(V·s), and is shown in the diagram as a grey, dashed line.

The strong dependence of the Stern potential on the parameter p is also visualised in Fig. S3, again using the parameter of the sample PVC@0.20. The intersections of the curves with the measured dimensionless electrophoretic mobility (grey, dashed line) characterise possible values for the dimensionless Stern potential.

4. Results and Discussion of the Samples PU@0.07 and PBAMM@0.10

All results for the samples PU@0.07 and PBAMM@0.10 are compiled in Table S6. Details on the determination are provided in the main article.

Table S6: Compilation of κa values, zeta potentials ζ and Dukhin numbers Du [a)]

material	κa_{CVI} [b)]	κa_{ELS} [b)]	$\zeta_{\mathrm{CVI}}^{\mathrm{d+s}}$ in mV	$\zeta_{\mathrm{ELS}}^{\mathrm{d}}$ in mV	$\zeta_{\mathrm{ELS1}}^{\mathrm{d+s}}$ in mV	$\zeta_{\mathrm{ELS2}}^{\mathrm{d+s}}$ in mV	$Du^{\mathrm{d+s}}$ [b)]	Du^{d}	$\frac{Du^{\mathrm{s}}}{Du^{\mathrm{d}}}$
PU@0.07	13.5 ±2.4	11.4	-51.4 ±2.0	-54.8 ±1.4	-60.1 ±1.5	-59.3 ±1.5	0.348	0.122	1.85
PBAMM@0.10	31.9 ±8.1	16.3	-57.7 ±3.2	-72.4 ±3.1	-68.8 ±2.9	-63.2 ±2.7	0.114	0.067	0.70

[a)] The subscript refers to the measurement technique used (the number in the subscript indicates solutions obtained using different evaluation models: 1 refers to the modified OHW theory and 2 to the DS theory) and the superscript to the surface conductivity contribution considered. $Du^{\mathrm{d+s}}$ is determined experimentally on the basis of MWO theory and covers both the contribution of the diffuse layer (Du^{d}) and that of the stagnant layer (Du^{s}). Du^{d} is calculated according to eq 9 on the basis of the $\zeta_{\mathrm{CVI}}^{\mathrm{d+s}}$ values and Du^{s} as the difference of $Du^{\mathrm{d+s}}$ and Du^{d}. Calculated standard deviations are absolute values.

[b)] Values determined in our recent publication. For further details, refer to [1].

4.1. Estimation of κa Values

In contrast to the other samples of the study, PU@0.07 and PBAMM@0.10 show clear signs of non-equilibrium dilution, as can be seen from the differences in the zeta potentials in Fig. S1. This is also indicated by the relatively high standard deviation of the κa_{CVI} values (>15 %). However, the κa_{CVI} and κa_{ELS} values of PU@0.07 are close to each other, suggesting that the electrolyte content could have a comparable impact on the electrical double layer in both cases. This is not expected for PBAMM@0.10, as the κa_{CVI} and κa_{ELS} values differ considerably. It should also be noted that PU@0.07 has the lowest κa value of all polymer dispersions investigated in this study. With a value of just over 10, it is no longer possible to consider the thickness of the diffuse layer to be very small compared to the particle size.

4.2. Zeta Potential Determination and Dukhin Number

Limited sample quantities were available for the dispersions PU@0.07 and PBAMM@0.10. Therefore, sample dilution was performed as described in section 3.2 of the main article with an electrolyte solution of known concentration, neglecting the original electrolyte content. This led to non-equilibrium dilution effects, which must be considered in the discussion of the following results.

In the case of PU@0.07, we found quite similar values for $\zeta_{\mathrm{ELS}}^{\mathrm{d}}$ (-54.8 mV), $\zeta_{\mathrm{CVI}}^{\mathrm{d+s}}$ (-51.4 mV), $\zeta_{\mathrm{ELS1}}^{\mathrm{d+s}}$, (-60.1 mV) and $\zeta_{\mathrm{ELS2}}^{\mathrm{d+s}}$ (-59.3 mV). This seems a bit surprising, since the contribution of the SLC is noticeable compared to that of the SLC ($Du^{\mathrm{s}}/Du^{\mathrm{d}}$ = 1.85). However, in addition to the effects due to non-equilibrium dilution, we have to bear in mind that the thickness of the diffuse layer for this sample is already quite large in relation to the particle size (κa = 11.4). Therefore, the validity of the evaluation methods may no longer be strictly given and $\zeta_{\mathrm{CVI}}^{\mathrm{d+s}}$ and $\zeta_{\mathrm{ELS2}}^{\mathrm{d+s}}$ values should only be considered with caution. The zeta potentials obtained using the original and the modified OHW theory, on the other hand, are very reliable as they are valid for $\kappa a \geq 10$. Due to the relatively low zeta potential and a rather low contribution of DLC to the surface conductivity, $\zeta_{\mathrm{ELS,H}}$ (-51.9 mV) and $\zeta_{\mathrm{ELS}}^{\mathrm{d}}$ (-54.8 mV) differ only slightly from each other. However, the modified OHW theory allows the SLC contribution to be taken into account. This results in a higher and more realistic value for $\zeta_{\mathrm{ELS1}}^{\mathrm{d+s}}$ (-60.1 mV).

The situation is a little different for PBAMM@0.10. The κa values show that the electrolyte concentration decreases significantly upon dilution. This pronounced non-equilibrium dilution has a considerable influence on the thickness of the diffuse layer. It is much larger at low particle volume fractions ($\kappa a_{\mathrm{ELS}} = 16.3$) compared to high ones ($\kappa a_{\mathrm{CVI}} = 31.9$). Consequently, the compression of the diffuse layer causes a decrease in the zeta potential. Hence, it is reasonable that the magnitude of the zeta potential obtained with the CVI method ($\zeta_{\mathrm{CVI}}^{\mathrm{d+s}} = -57.7$ mV) is significantly lower than that determined by the ELS method ($\zeta_{\mathrm{ELS}}^{\mathrm{d}} = -72.4$ mV and $\zeta_{\mathrm{ELS1}}^{\mathrm{d+s}} = -68.8$ mV). Due to the moderate contribution of SLC to the surface conductivity ($Du^{\mathrm{s}}/Du^{\mathrm{d}} = 0.70$), the values of the original and the modified OHW theory should be quite similar. In fact, the differences are small and can probably be attributed to the non-equilibrium dilution, which also affects the determination of the $Du^{\mathrm{d+s}}$ number. Moreover, the violation of the validity criterion of the DS theory becomes obvious from the implausible calculated potentials (cf. Table S4), since the absolute value of the Stern potential ($\psi_{\mathrm{d,ELS2}}^{\mathrm{d+s}} = -45.6$ mV) is much lower than that of the zeta potential ($\zeta_{\mathrm{ELS2}}^{\mathrm{d+s}} = -63.2$ mV).

Figure S4 finally depicts the zeta potentials measured by the CVI method as a function of the particle volume fraction, taking into account the $Du^{\mathrm{d+s}}$ values. Obviously, the values for PU@0.07 and PBAMM@0.10 show a slight but systematic trend, which is presumably due to the noticeable non-equilibrium dilution. Some values are therefore neglected in the calculation of the average zeta potential value $\zeta_{\mathrm{CVI}}^{\mathrm{d+s}}$. However, this effect is rather small so that the results are still quite reliable.

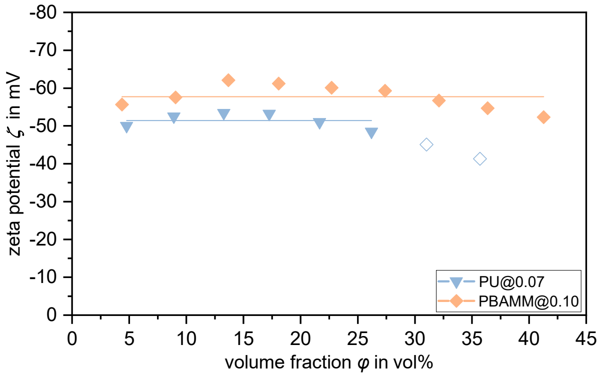

Figure S4. Zeta potential ζ measured by CVI method as a function of particle volume fraction φ for samples PU@0.07 and PBAMM@0.10. The Dukhin number $Du^{\mathrm{d+s}}$ determined experimentally on the basis of the MWO theory was used. Filled data points were considered for the calculation of the average zeta potential value $\zeta_{\mathrm{CVI}}^{\mathrm{d+s}}$ (shown as solid line and listed in Table S6), whereas unfilled data points were neglected. The pH values of the samples were 8.8 for PU@0.07 and 8.6 for PBAMM@0.10. The PU and PBAMM samples were directly diluted to achieve a nominal KCl concentration of 10 mmol L^{-1} in the dispersion medium, neglecting the original electrolyte content.

5. References

(1) Frangenberg, M., Schmidt, A. M., Wilkens, J. Impact of surface conductivity on the zeta potential determination of concentrated aqueous polymer dispersions using electroacoustics and electrokinetic standard models. *Colloid Polym. Sci.* **2024**, *302*, 1801–1813.

(2) Van Der Put, A.G, Bijsterbosch, B. Electrical conductivity of dilute and concentrated aqueous dispersions of monodisperse polystyrene particles. Influence of surface conductance and double-layer polarization. *J. Colloid Interface Sci.* **1980**, *75*, 512–524.

(3) Delgado, A. V., González-Caballero, F., Hunter, R. J., Koopal, L. K., Lyklema, J. Measurement and interpretation of electrokinetic phenomena. *J. Colloid Interface Sci.* **2007**, *309*, 194–224.

(4) International Organization for Standardization. *Colloidal systems - Methods for zeta-potential determination - Part 1: Electroacoustic and electrokinetic phenomena*, 2012(13099-1:2012-06). https://www.iso.org/standard/52807.html.
